# Some Clinical Aspects of Granular Kidney

**Published:** 1899-03-04

**Authors:** Samuel West


### HOSPITAL CLINICS AND MEDICAL PROGRESS.

### SOME CLINICAL ASPECTS OF GRANULAR KIDNEY,

Abstract of the Lettsomian Lectures delivered before the Medical Society of London by SAMUEL WEST, M.D., F.R.C.P.

THE second of these lectures was devoted by Dr. West to a consideration of the two symptoms, albuminuria and albuminuric retinitis. Albuminuria in granular kidney is usually small in amount and is not constantly present; in fact, it may be absent for long periods, and even, it is said, altogether, though this is hard to prove. When present its amount may vary greatly, for although usually small in quantity, it may be as much as half, or more; but this generally depends on some accidental complication, such as current nephritis or failure of the heart. When, then, the presence of albumen in the urine is the chief symptom, one has to decide from other signs whether granular kidney is the cause or not; and when, as often happens, the patient appears to be in an ordinary condition of health, we may seem to have to do with the so-called physiological or functional albuminuria.

Albuminuria has been produced experimentally in perfectly healthy persons by excessively albuminous diet, by violent muscular exercise, by cold bathing, and exposure to cold; but even then there is probably some other factor necessary, for it is not produced in every person with equal ease. Again, in an apparently healthy person albumen may be found occasionally under certain circumstances, as, for example, on rising in the morning or after food. There still remains, however, a group of cases in which albuminuria is present with apparently nothing whatever in the previous history or the actual condition of the patient to explain it. The choice of a term by which these cases can be described is difficult, but "albuminuria in the apparently healthy" seems to be the best.

Dr. West goes carefully into the question of the frequency with which this form of albuminuria is met with, but the results obtained by the different observers quoted vary so greatly that, as he says, "there must be a fallacy somewhere." It seems clear, however, that the condition is far from uncommon, especially in youth.

If, then, we turn to the question of the significance of this albuminuria in the apparently healthy it would seem that, if albuminuria is so much more common before than after 25 years of age as is shown to be the case, many of the cases can have no very serious significance—in other words, they must get well. This, indeed, we know from direct observation.

Granting, however, that many, perhaps the majority, ultimately recover, there remains another group in which the patients continue to pass albumen for many years and yet remain in good health, and a third in which signs of renal disease ultimately develop.

Again, it has to be pointed out that maintenance of health for many years is not incompatible with damaged kidneys ending in premature death. So that it is by no means certain that some of the cases which in youth appear purely functional may not be the beginnings of organic disease.

In adult life—that is after about 25 years of age—the significance of albuminuria becomes more serious, for the chances of renal disease rapidly increase, and careful attempts to trace out the after history of patients applying for insurance who, although otherwise apparently healthy, have shown traces of albuminuria, tend to show that such cases have a mortality rate for kidney disease much above the normal.

We may thus conclude that for the ages at which life insurances are generally effected—that is from 25 years upwards, the presence of even a trace of albumen is of considerable significance owing to the difficulty of excluding the possibility of its being due to commencing granular kidney.

The conclusions then to which Dr. West arrives in regard to this subject are that the so-called physiological albuminuria is always pathological even if not always renal, when the amount of albumen is more than the merest trace, and probably pathological even in those cases when there is but a trace, and no cause obvious. In this respect it is interesting to recall the experience of Eales, who, out of 14 cases sent him as physiological albuminuria, found no fewer than five with marked albuminuric retinitis, showing that what he had to do with was not physiological albuminuria at all, but well advanced granular kidney. Every case, therefore, must be minutely examined. If albuminuria exist alone it may possibly be of no serious significance. But if renal derivatives are found as well as albumen it must almost necessarily be due to renal disease, and this is in all probability also the case, even if no renal derivatives are found, in patients in whom the arteries are thickened and the pulse tension high.

### ALBUMINURIC RETINITIS.

In its early stage and typical form, albuminuric retinitis is characteristic and pathognomonic—it occurs in no other condition except that of chronic renal disease. In the latter stages, however, and especially in the inflammatory form, it may closely resemble the optic neuritis met with in some brain affections; but in such cases its importance is not so great, for the diagnosis is generally clear on other grounds.

The question arises whether albuminuric retinitis occurs in other forms of kidney disease besides the granular kidney. In amyloid kidney Dr. West has not seen it, in acute parenchymatous nephritis it seems never to be met with; but it is stated to occur occasionally in chronic parenchymatous nephritis. In regard to the latter, however, it must be remembered that the kidneys may have been granular before the acute nephritis commenced.

The lesions in albuminuric retinitis consist in (1) white patches, (2) hæmorrhages, (3) exudations, and (4) inflammatory conditions. These generally develop in the order in which they are given above, and it is to be noted that the earliest and most characteristic of them are the white patches, which are to be found in the region of the macula, round which, in their typical and favourite form, they are arranged in a radiating fashion like the spokes of a wheel. But, early as the white patches are, they are preceded by and result from still earlier changes in the vessels, which are of the nature of obliterative arteritis leading to inflammatory degeneration of the structures supplied. These changes are really the early changes of granular kidney. In regard to the question, Which are the primary changes, the vascular or the renal? the fact that by the time these changes are to be found in the eye the kidneys are so often already granular, would seem to show that the vascular changes are late, considering how early the changes in the retina could be detected by the ophthalmoscope if they were present.

The early lesions of albuminuric retinitis produce no defects of vision, and even in later stages it is very remarkable how extreme the eye changes may be without a complaint being made of impairment of sight. As a rule we may say that when patients complain of defect of vision the albuminuric retinitis is already very far advanced, and when vision once begins to fail it often fails rapidly, so that in two or three weeks it may be completely lost. The significance of albuminuric retinitis is always grave. It is impossible to give figures, because the condition is so often not discovered until it has lasted for some time, but speaking generally the duration of life after the discovery of albuminuric retinitis is short—not many months.

